# Enabling alloy screening via multi-material printing using a production PBF-LB machine

**DOI:** 10.1007/s40964-026-01939-7

**Published:** 2026-08-29

**Authors:** Victor M. Villapun, Luke N. Carter, Daisy Rabbitt, Ming Huang, Daniel Wilmot, Jonathan D. Stevenson, Richard J. M. Hague, Sophie C. Cox

**Affiliations:** 1 School of Mechanical Engineering, University of Birmingham, Birmingham, UK; 2 School of Chemical Engineering, University of Birmingham, Birmingham, UK; 3 Department of Engineering and Design, University of Sussex, Brighton, UK; 4 School of Metallurgy and Materials, University of Birmingham, Birmingham, UK; 5 Royal Orthopaedic Hospital, Birmingham, UK; 6 Centre for Additive Manufacturing, University of Nottingham, Nottingham, UK

**Keywords:** Additive manufacturing, Laser powder bed fusion, Multi-material, Alloy discovery, High-throughput techniques, Medical devices

## Abstract

The development of novel functional alloys is often constrained by the onerous and costly discovery to validation process. Herein, we present a set-up to enable multi-material production of specimens through in-situ alloy formation using a production laser-based powder bed fusion (PBF-LB) printer. Multi-material samples were achieved through the combined use of a reduced build volume unit, to minimise material requirements (~ 16% powder volume of full build volume), and custom spacers to screen a Ti-Cu biomedical alloy design space allowing for identification of performance thresholds. Multi-material specimens were designed from 0 to 20 wt% Cu in 5% increments and rapidly assessed using high-throughput methods for mechanical and biological properties. Specifically, microhardness and non-destructive ultrasound analysis showcased a significant enhancement in hardness, from 199.24 ± 5.97 to 428.53 ± 26.75 HV, and reduction in longitudinal modulus, from 166.59 ± 4.95 to 148.37 ± 7.54 GPa, as copper content increased. Simultaneously, confocal imaging of pre-osteoblasts (MC3T3s), mesenchymal stem cells (hMSCs), and gram-positive *Staphylococcus aureus* bacterium after 24 h of contact, suggested 10% Cu as the breakpoint to maintain cytocompatibility and antimicrobial efficacy. Designed Ti-10Cu was identified as the region of interest for further alloy refinement, demonstrating the ability of this screening system to narrow down a material design space according to critical functional properties. This proof-of-concept study presents a feasible alloy discovery tool that utilises a production PBF-LB system without any machine modification.

**Graphical abstract:**

**Figure:**
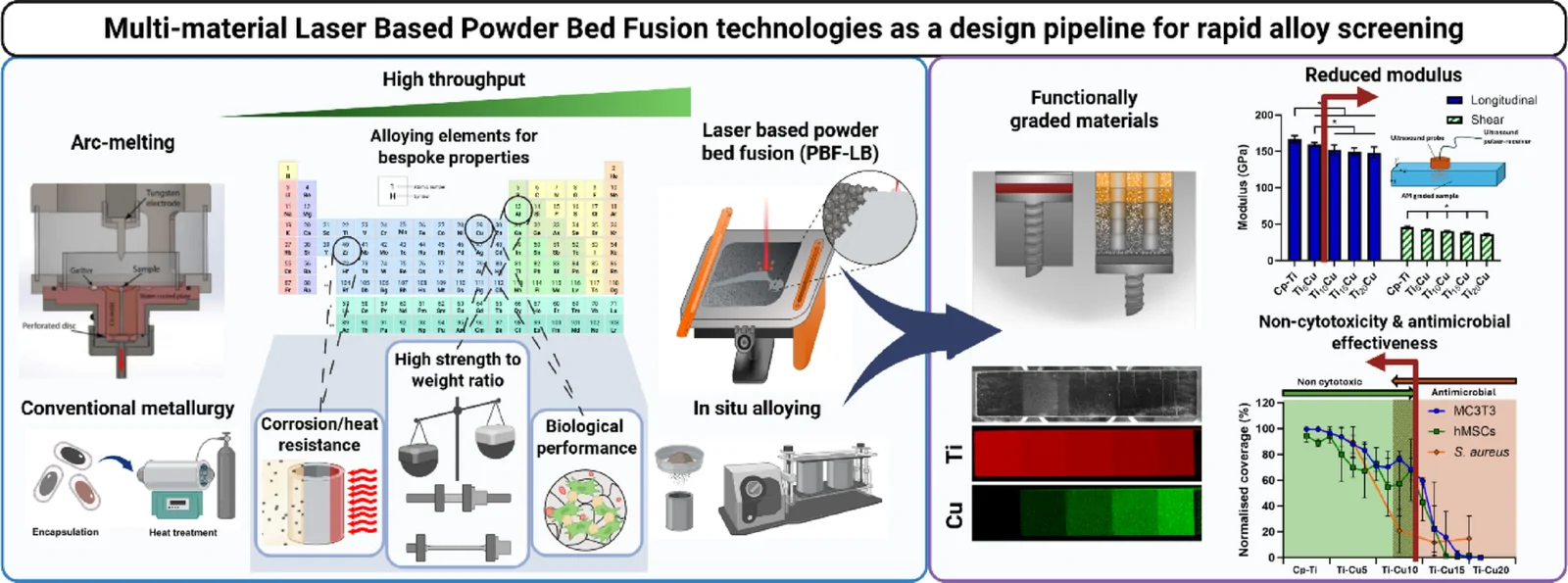

## Introduction

Technological advancement is intrinsically linked to the development and application of novel materials [1]. For instance, aerospace and fusion reactors are constrained by high strength to weight ratio, wear, corrosion, heat or radiation resistant structural materials [2, 3], while healthcare presents specific needs of mechanical and corrosion resistance balanced with biological functions [4]. Although several novel alloys have been developed (e.g. high entropy alloys) [5, 6], the discovery phase involves prolonged experimental testing and validation. Alloy development typically starts with a core element, or binary/ternary alloying system further supplemented to fine tune specific secondary properties. Some alloys such as α + β Ti-6Al-4V (Ti64) are relatively simple with aluminium reducing density, and vanadium, enhancing toughness and formability [7, 8]. However, single crystal nickel super alloys can have between 5 and 10 constituents for γ′ stabilisation or solid solution, grain boundary strengthening (e.g. CMSX-4, Ni_60.7_Cr_7_Co_9_Mo_0.6_W_6_Al_5.6_Ti_1_Ta_7_Hf_0.1_Re_3_ wt%) [9, 10]. At research scales, traditional approaches focus on iterative and incremental single elemental additions with vacuum arc-melting, induction melting or powder metallurgy processes, enabling the production of 1–10 gram sized ingots per day [4, 11, 12]. This “one alloy at a time” approach combined with limited sample production and a vast range of alloying elements results in a time consuming and costly process. For example, Ti64 introduced in the late 1950s as an orthopaedic metal is still the most widely used material despite having a dissimilar modulus to native bone (112 GPa versus 17.8–28 GPa, respectively), which is a leading cause of implant failure [4]. Low modulus alternatives exist (e.g. 55 GPa for Ti-Nb-Ta-Zr), however, with 30–40 identifiable elemental candidates for a bioinspired Ti-based alloy, there is a need for effective discovery screening processes, for which additive manufacturing (AM) may offer opportunities.

The capability of layer-by-layer AM technologies to produce complex light-weight geometries has supported aerospace, automotive and energy applications while customised implants are delivering valuable healthcare benefits. Of the metal AM processes, laser-based powder bed fusion (PBF-LB) and direct energy deposition (DED) are the most explored technologies, however, the higher resolution (50 vs. 500 μm) of PBF-LB, generally makes it of greater industrial relevance capturing 50.7% of the market in 2024 [13–15]. PBF-LB makes use of high-powered laser(s) to selectively melt metallic powder layer-by-layer. The possibility to premix elemental powders to form custom alloys in-situ via PBF-LB is expanding as a material development tool. This in-situ alloying approach has been successfully employed to explore alloy systems, microstructural optimisation or reinforcement of existing alloys [16–18].

While PBF-LB has historically been restricted by the use of a single feedstock powder mix per print [19, 20], multi-material PBF-LB has recently enabled simultaneous use of dissimilar stock powders in a single print [21–23]. Selective Powder Deposition (SPD) from Aerosint [24] is the main commercial multi-material PBF-LB option, involving substitution of blade recoaters with rotating powder patterning drums to produce multi-material layers with a resolution of 300 μm. Several research groups have also reported alternative in-house hardware modifications to create multi-material PBF-LB platforms. One approach by Demir et al. increased the number of hoppers to control the delivery of Fe/Al-12Si to an in-situ mixer supplying the recoater [25]. Conversely, other platforms make use of micro-vacuum systems to selectively remove powder from spread layers, followed by dispersion of two to three material powders through ultrasonic dispensers (e.g. 316 L and In718/Cu10Sn) [26]. Alternative approaches have modified the recoater, providing two reservoirs, e.g. In718 and 316 L, for full layer deposition [27]. While it is apparent that multi-material PBF-LB solutions are in the making, aside from the Aerosint technology these approaches require machine modifications [27–30]. Emphasis has also been placed on dual material production to achieve surface/bulk targeted properties [16, 28, 31], limiting the potential of multi-material PBF-LB as an alloy screening tool.

Development of an alternative approach to leverage standard production PBF-LB machines to manufacture multi-material alloys would open up the use of AM as a material screening method. Such a pipeline may be especially valuable in healthcare where there has been relatively limited sector-specific functional design, with the gold standard Ti64 originally repurposed from aerospace [4]. For instance, copper alloying has gained traction in the research community as a desirable biologically active element due to its antimicrobial activity, further amplified by potential effects on mineralisation, vascularisation and immunomodulation [32]. More broadly, copper alloying in titanium-based metals could promote equiaxial growth and grain refinement [33, 34] that, in conjunction with thermal control, can limit PBF-LB microstructural anisotropy and enhance mechanical behaviour. These benefits have resulted in an increased number of manuscripts exploring its incorporation in titanium-based alloys either through conventional manufacturing (e.g. casting, arc-melting) [35–37] or more recently using PBF-LB [38–40] technologies. For instance, Yi et al. [35] and Ju et al. [38] have explored the role of copper content into Ti-Cu and Ti-6Al-4V-Cu, demonstrating the enhancement in hardness caused by increased copper content due to microstructural refinement. On the other hand, other authors have focused on their biological potential showcasing the impact of copper content in cytotoxicity and antimicrobial properties [37, 41, 42], suggesting 5–10 as an area of interest to provide safe and functional materials. Nevertheless, discrepancies on its biological mode of action, optimal composition, microstructure and processing complexities (i.e. laser reflectivity of Cu) still exist, limiting clinical translation of titanium copper alloys [37, 39].

**Fig. 1:**
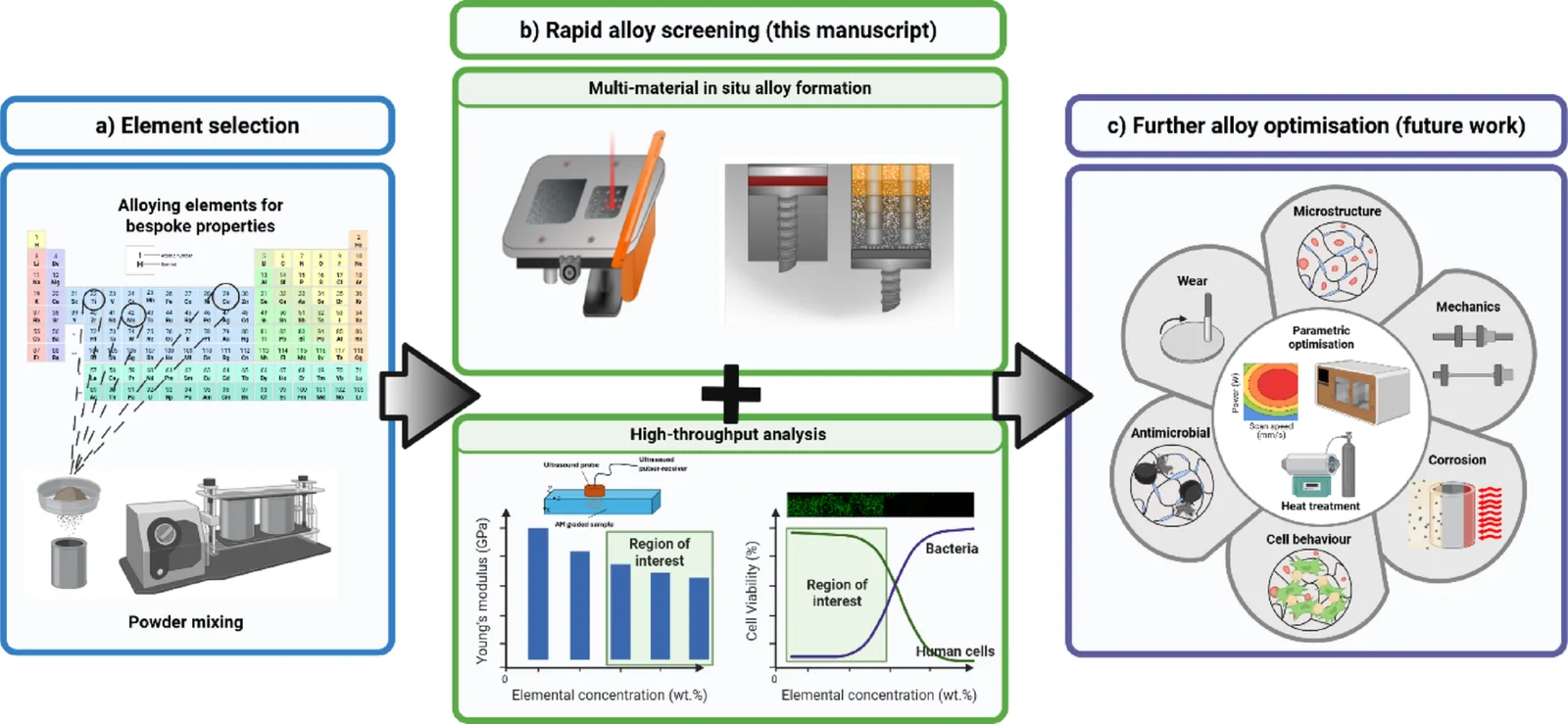
Proposed alloy screening pipeline through the use of (**a**) in-situ alloying, (**b**) multi-material PBF-LB and high-throughput techniques to enable (**c**) focused alloy optimisation

Herein, we propose a pipeline for streamlined screening of novel alloys to identify threshold breakpoints for particular applications to facilitate material selection and further optimisation through combined use of a production PBF-LB system operated with a reduced build volume (RBV) and a bespoke dosing kit (Fig. 1). Using Ti-Cu as an example antimicrobial alloy system with preexisting compositional trends, a multi-material designed with 0–20 wt% Cu was produced and analysed using SEM-EDX, XRF and XRD. Mechanical properties were evaluated with non-destructive ultrasonic probing and microhardness. Biological analysis through confocal imaging of murine MC3T3 preosteoblasts, human mesenchymal stem cells (hMSCs) and gram-positive *Staphylococcus aureus*, revealed designed Ti-10Cu as a compositional breakpoint balancing antimicrobial effectiveness with non-cytotoxicity. In summary, this manuscript reveals the possibility to retrofit a PBF-LB system enabling alloy design space screening through multi-material printing and assessment of key properties supporting identification of promising design regions for further refinement and testing. Coupled with high-throughput validation methods, the proposed approach expands the versatility of multi-material PBF-LB to support materials discovery.

## Experimental

### PBF-LB of multi-material samples

All samples were produced using a RenAM 500 S (Renishaw PLC, UK) equipped with a reduced build volume unit (RBV, Renishaw PLC, UK). Cp-Ti (AP&C, Canada) and pure copper (Eckart, UK) feedstock powder, 12–83 μm and 4–50 μm, respectively, were mixed on a roller (Evans Lapidary, UK) for 6–8 h under argon atmosphere. Ti-XCu ratios, X = 5, 10, 15 and 20 wt%, were alloyed in-situ using a single parameter set. Parameters were selected based on previous studies showing good consolidation regardless of blended powder composition [43]: layer thickness 30 μm, laser power 100 W, scanning speed 600 mm/s (90 μm point distance and 150 µs exposure time), hatch distance 90 μm, spot size 80 μm and single contour.

**Fig. 2:**
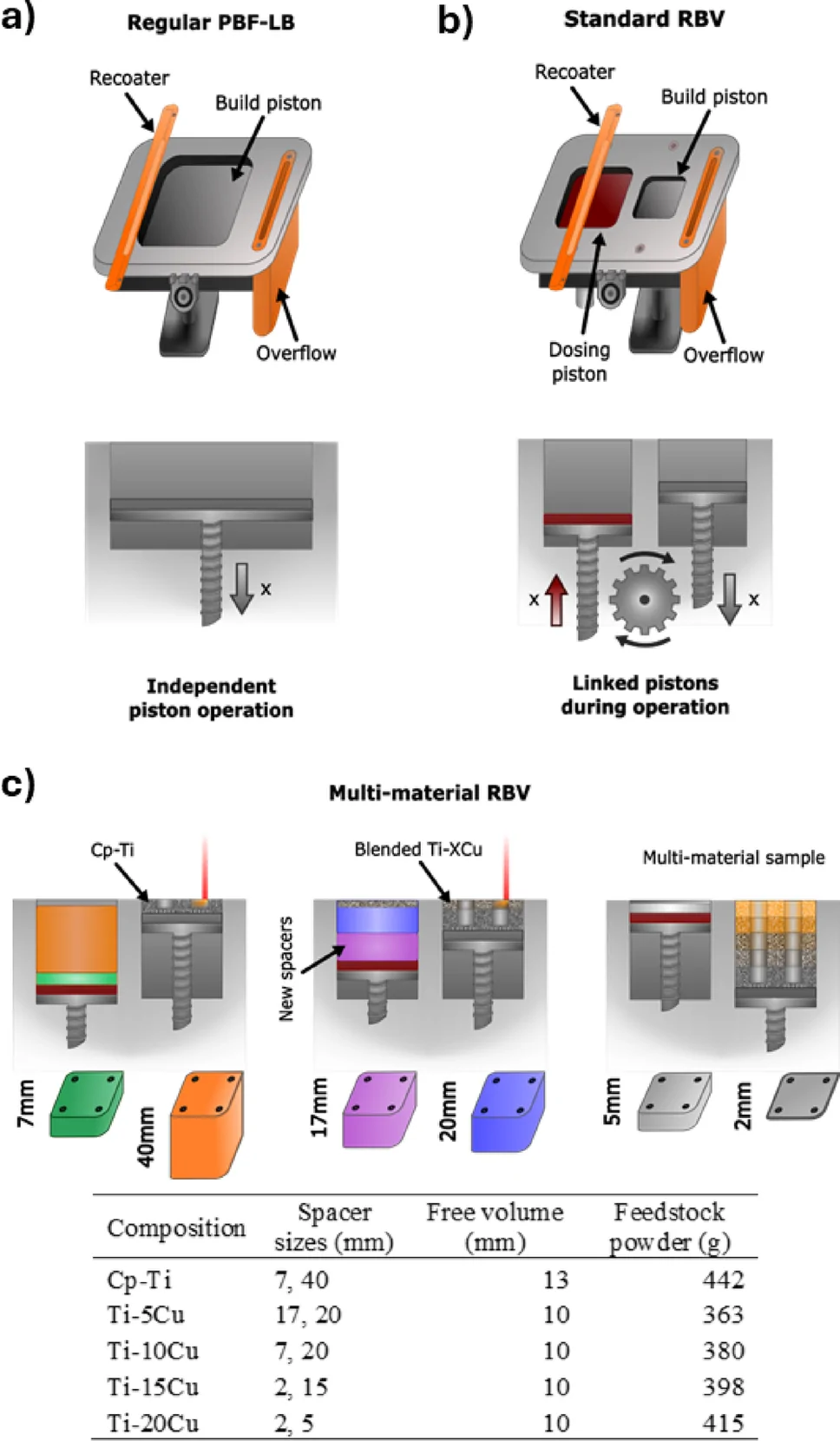
Depiction of (**a**) conventional PBF-LB systems with an independent build platform, contrasting with (**b**) conventional RBVs linking together a dosing and build piston which was further modified through (**c**) in-situ alloying and custom-made spacers to enable multi-material Ti-Cu sample production for high-throughput analysis

Typically, the Renishaw PBF-LB machine used in this study employs a single piston to control the build volume, with powder supplied by a separate system (Fig. 2a). In contrast, the single machine piston simultaneously operates both build volume platform and powder supply area with a 1:1 volume ratio within the RBV unit (Fig. 2b). Reduction of the build plate from 250 × 250 mm to 100 × 100 mm in the RBV decreases powder volume from 10–20 kg to 0.5–1 kg.

To enable multi-material production, the RBV was coupled with supply volume spacers manufactured via Fused Deposition Modelling (FDM) in polylactic acid, PLA, to further reduce the amount of feedstock powder and control its height to produce 10 mm sections of each alloy (Fig. 2c). Firstly, several spacers were stacked leaving a 13 mm gap (3 mm for supports and 10 mm coupons) that was filled with pure Cp-Ti powder calculated as 70% of the available volume due to free space between particles. After the first composition was processed, a series of new spacers were placed on the RBV, leaving a 10 mm gap, then the next alloy composition (e.g. Ti-5Cu wt%) was added and the process continued. By repeating this step, it was possible to produce two proof-of-concept multi-material samples for streamlined alloy screening. One was employed for non-destructive mechanical analysis while all remaining tests were conducted on the second multi-material sample with technical repeats performed across assays.

### Additive manufacturing process monitoring

To monitor variations in processing conditions, a Renishaw Additive Manufacturing Process Monitoring (AMPM) system was used to collect infrared emission data from photodiode signals corresponding to the ‘melt-pool’ (1090–1700 nm) and ‘plasma’ (700–1040 nm). 43 layers from the mid-region of each composition were identified on the central 4 × 4 mm X/Y region of the specimen to ensure stabilisation of the build process while obtaining a wide range of hatch vectors due to the 67° rotation between each layer [44]. The mean of all datapoints was taken as a representative value for the AMPM signal measured.

### Scanning electron microscopy (SEM)

Surface analysis was performed using a EVO10 SEM (Zeiss, Germany) at 20 kV equipped with a x-act energy-dispersive X-ray (EDX) detector (Oxford Instruments, UK). All multi-material builds were serially ground (240, 1200 and 4000 grit size), mirror polished with non-activated colloidal silica, cleaned in ethanol (20 min twice in an ultrasonic bath) and air dried.

### Microstructural and chemical characterisation

X-ray Diffraction (XRD) was performed on a AXRD benchtop system (Proto Manufacturing, USA), between 2Ɵ = 0–90°, a step size of 0.0149° at 30 kV, 20 mA, a copper line focus X-ray tube and a nickel kβ absorber (0.02 mm; Kβ = 1.392250 Å). Background was removed using CrystalDiffract version 7.1.1 (CrystalMaker Software Ltd, USA) with scans normalised using the peak displaying the strongest signal (134.4, 72.4, 44.1, 32.6 and 48.6 counts/s for Cp-Ti and designed Ti-5Cu, Ti-10Cu, Ti-15Cu and Ti-20Cu, respectively). Chemical evaluation was complemented with X-ray fluorescence (XRF) using a M4 Tornado and ESPIRIT, 2015, software (Bruker, Berlin, Germany) under vacuum, a distance between points of 25 μm, serpentine pathway and 5 repeating cycles.

### Microhardness

Vickers hardness, HV0.1, was carried out with 100 g in a Wilson VH1202 microhardness system (Buehler, UK). Ten indentations, five each for differently shaded precipitates, were obtained per alloy with manual adjustments performed when the software (DiaMet, Buehler, UK) failed to accurately locate indent corners.

### Longitudinal and shear moduli

**Fig. 3:**
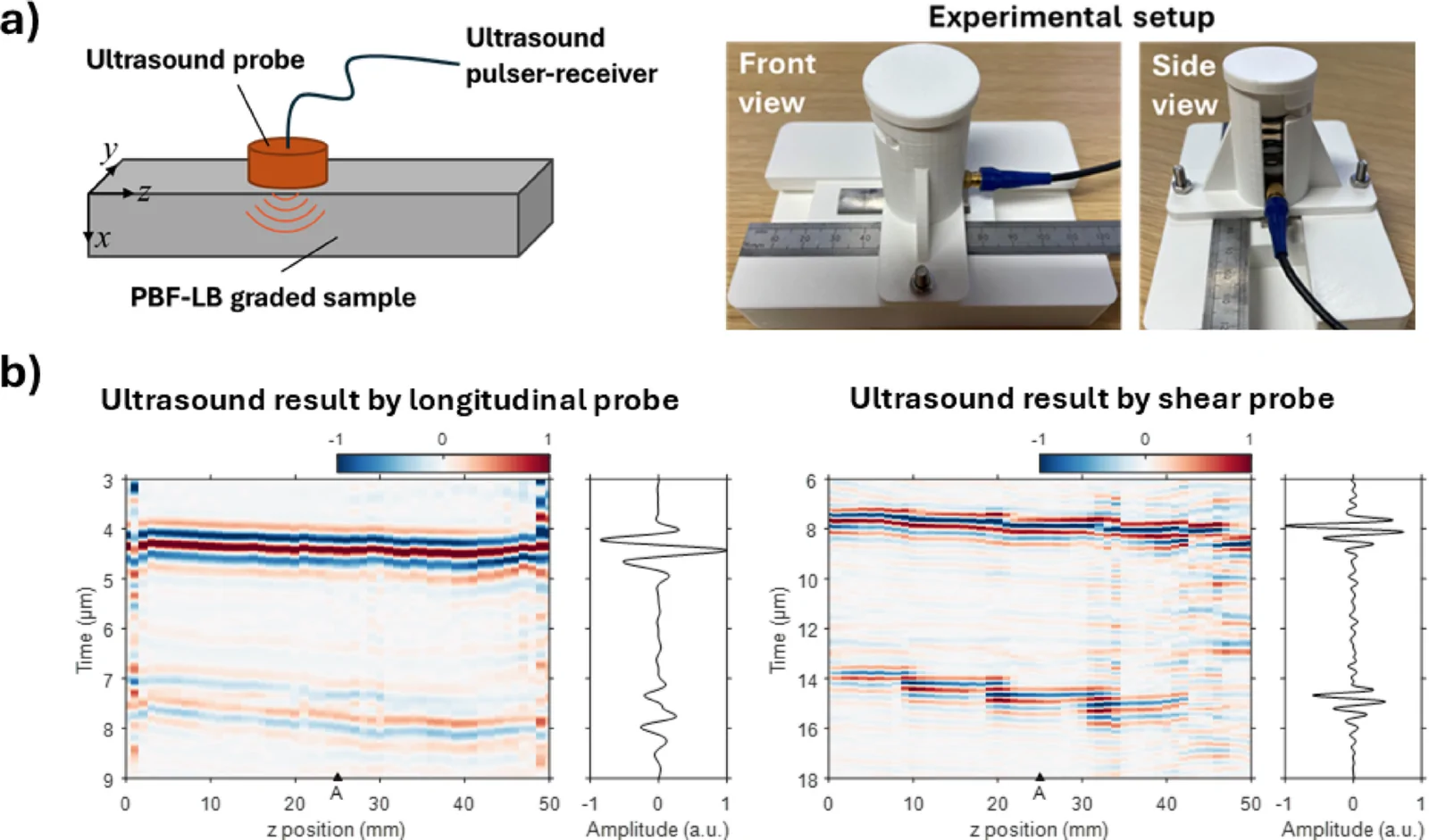
Ultrasonic characterisation of the longitudinal and shear moduli for the Ti-Cu multi-material sample indicating (**a**) experimental setup and (**b**) ultrasound signal maps from longitudinal and shear probes with example signals

The longitudinal and shear moduli were characterised using a non-destructive ultrasonic technique (Fig. 3a). Longitudinal and shear wave measurements were performed using 2.25 MHz probes (0.5-inch diameter, V106 and V154, Waltham, MA, USA), with wave generation and signal acquisition controlled by a pulser-receiver (Handyscope HS5, Tiepie, Netherlands). The acquired signals were plotted as colour maps (Fig. 3b) for the longitudinal and shear wave cases, exhibiting clearly identifiable wave arrivals with a high signal-to-noise ratio (> 30 dB) at all measurement locations. At each measurement location, the time-of-flight $\:{\Delta\:}t$ was extracted and the longitudinal $\:{V}_{L}$ and shear $\:{V}_{S}$ wave velocities were calculated as $\:{V}_{L,S}\:=\:2d/{\Delta\:}t$, where * d* is the sample thickness. The spatially resolved longitudinal and shear moduli were determined from $\:M=\:\rho\:{V}_{L}^{2}$ and $\:G=\:\rho\:{V}_{S}^{2}$, respectively, where $\:\rho\:$ is the density [45, 46].

### In-vitro evaluation

Eukaryotic cellular interactions were assessed in-vitro using murine pre-osteoblast cells (MC3T3, P14) and human mesenchymal stem cells (hMSCs, P5). Before seeding, samples were disinfected for 30 min in pure ethanol and UV light followed by inoculation with 2 × 10^4^ MC3T3 cells/cm^2^ or 1.5 × 10^4^ hMSCs cells/cm^2^ and incubation for 10 min (37 °C and 5% CO_2_) to enable early cell attachment. Then, Dulbecco’s modified Eagle’s medium, DMEM, (10% FBS, 4.5 g/L D-Glucose, L-glutamine, and 1% penicillin/streptomycin) or DMEM-GlutaMax (10% FBS, 4.5 g/L D-Glucose and 1% penicillin/streptomycin) was added for MC3T3 or hMSCs, respectively, and samples cultured at 37 °C and 5% CO_2_ for 24 h.

Cell survival was assessed using Live/Dead staining with 200 µL/cm^2^ of a 5 mM calcein-AM (VWR International, USA) and 1.5 mM propidium iodide (Thermo Fisher scientific, USA) solution. After 30 min of incubation, each substrate was washed with DPBS, and imaged (EVOS M5000, Invitrogen, USA) using a x4 magnification lens.

To complement cytotoxicity assays, antimicrobial effectiveness was evaluated with, *Staphylococcus aureus*, Newmann strain. Briefly, sterilised surfaces were placed inside a petri dish containing DPBS wetted tissue. An overnight culture of *S. aureus* in Mueller Hinton broth was diluted to ∼10^3^ CFU/mL, inoculated with 200 µL/cm^2^ and cultivated at 37 °C for 24 h.

For confocal fluorescence imaging, the multi-material was washed with DPBS and stained with 200 µL/cm^2^ of a SYTO^®^ 9 and propidium iodide stain mixture (FilmTracer™, Invitrogen, USA). After 30 min incubation at RT, the sample was immersed in fresh DPBS and imaged in a ZEISS LSM 710 confocal microscope (Carl Zeiss GmbH, Germany) at ×20 magnification.

For both in vitro tests, coverage was calculated using ImageJ (National Institute of Health, version 1.53a) from at least 5 images per alloy using Cp-Ti for normalisation.

### Statistical analysis

SPSS Statistics (IBM Corp., Version 29.0.0.0, USA) was used to analyse the collected datasets. Firstly, Levene’s similarity of variances tests was performed. If similarity could not be rejected, and due to the use of technical repeats in the multi-material sample, a linear mixed model followed by Bonferroni’s post hoc with an alpha level of 0.05 was used while a rejection of similarity resulted in a Welch’s test and Games-Howell’s post hoc.

## Results

**Fig. 4:**
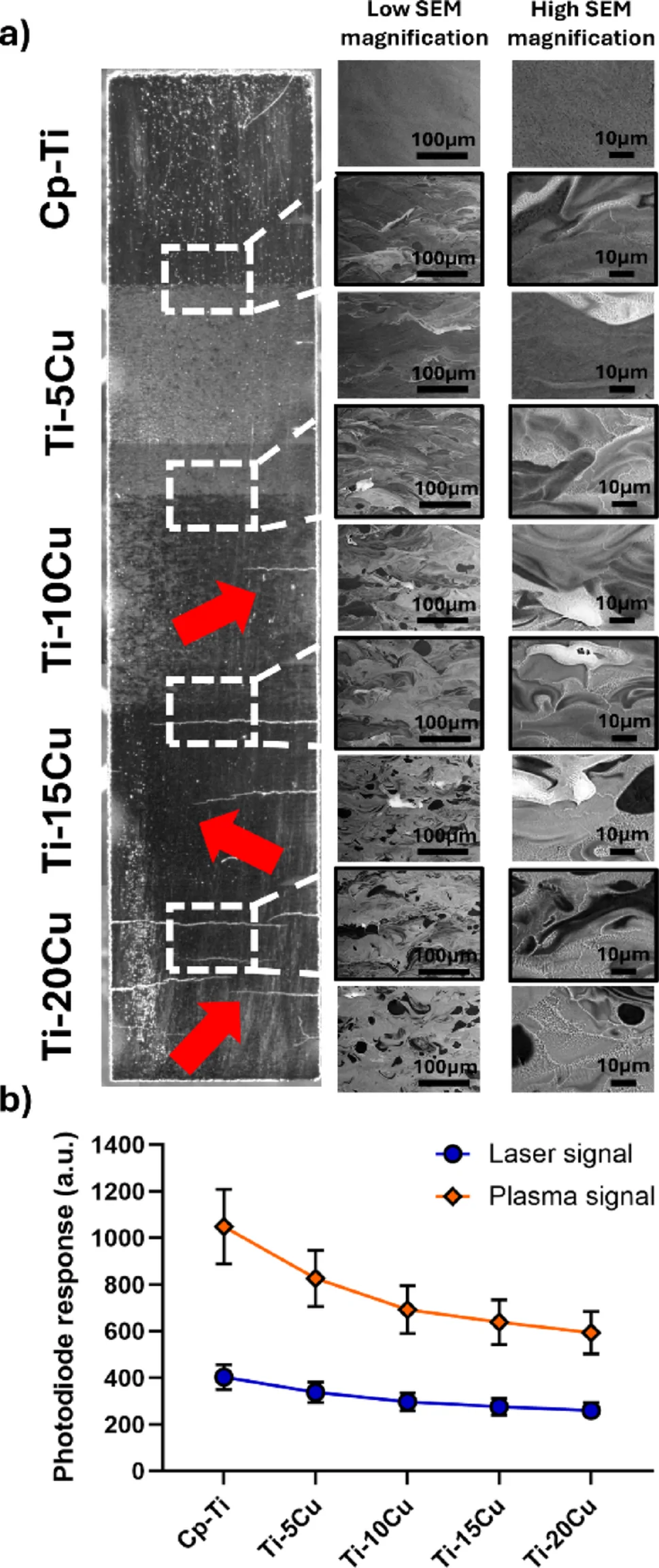
**(a)** Light micrograph and backscatter SEM images of the Ti-Cu multi-material sample with microstructural details at the centre and border, dashed rectangles, of each alloy (0–20 wt%), including thermal cracking (red arrows) and (**b)** AMPM photodiode response for laser and plasma signal. Captions indicate designed Ti-XCu alloys for X = 0–20 wt%

Multi-material Ti-Cu samples were produced with a reduced build volume (RBV) system coupled with a custom alloy dosing kit in a conventional PBF-LB machine (Fig. 2). Light micrographs revealed cracks parallel to the build plane as the copper content increased (Fig. 4a, red arrows). These may be caused by the presence of brittle intermetallics and liquation/thermal cracking resulting from variations in thermal properties between Cu and Cp-Ti such as thermal conductivity, 398 vs. 20 W/mK, respectively [47]. Additive Manufacturing Process Monitoring (AMPM) readings from the ‘melt-pool’ and ‘plasma’ (Fig. 4b) indicated composition directly affected melt pool area and/or temperature with increased copper content, in line with the work of Mosallanejad et al. [39]. At the same time, SEM imaging revealed the transition from a homogeneous to heterogeneous microstructure. By adding 5 wt% copper, it was already possible to discern areas of poorly incorporated clear, Cu-rich, and darker, Ti-rich, precipitates, more prominent as the titanium content decreased.

**Fig. 5:**
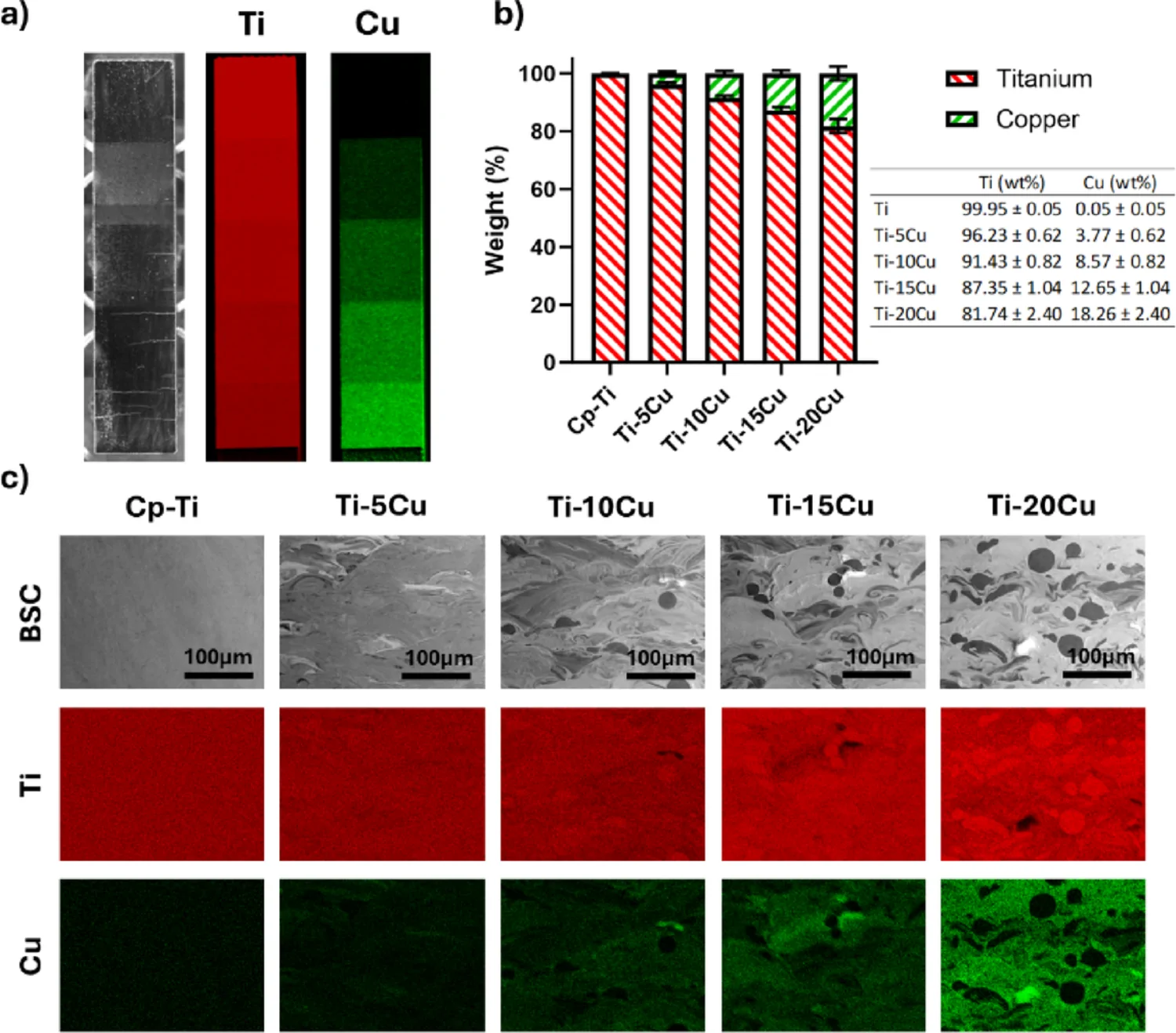
Chemical analysis of the multi-material sample with (**a**) X-ray fluorescence and (**b**) energy dispersive spectroscopy for quantitative evaluation of the targeted compositions including (**c**) elemental maps across alloying systems. Captions indicate designed Ti-XCu alloys for X = 0–20 wt%

The overall incorporation of copper in the multi-material sample is evident through XRF (Fig. 5a) and EDS analysis (Fig. 5b), nevertheless, differences from the objective alloy compositions could be observed, 3.77, 8.57, 12.65 and 18.26 wt% for the 5, 10, 15 and 20 wt% Ti-XCu ratios. EDS mapping of central areas of each alloy further confirmed material heterogeneity with potentially unmelted Cp-Ti particulates prominent in the 10, 15 and 20 wt% designed compositions (Fig. 5c), suggesting the need for individual composition parameter optimisation.

**Fig. 6:**
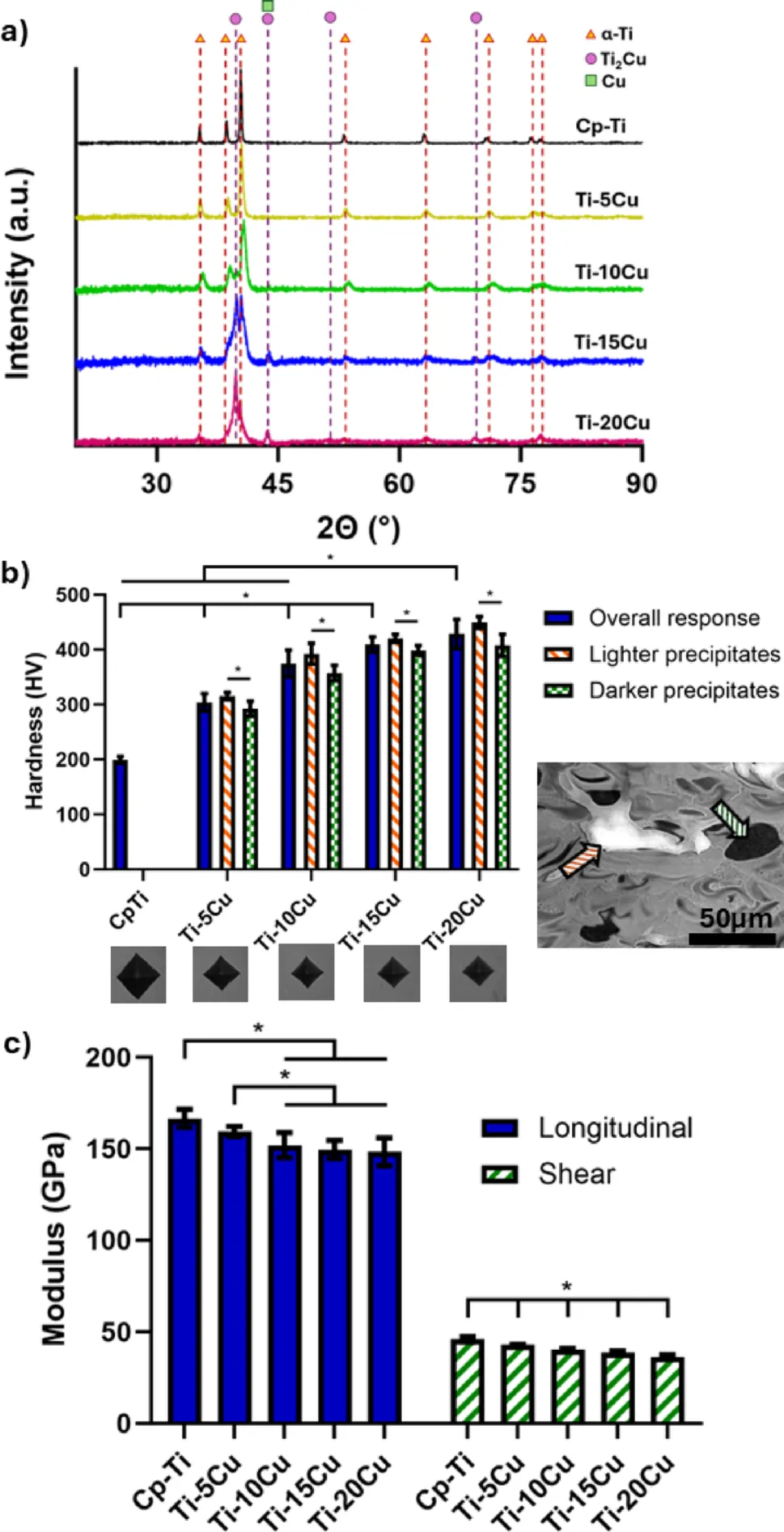
Variations in microstructure and mechanical properties of the multi-material samples showcased by **(a)** normalised X-ray diffraction, **(b)** Vickers microhardness and **(c)** longitudinal and shear moduli obtained through non-destructive ultrasonic analysis. Captions indicate designed Ti-XCu alloys for X = 0–20 wt%

Microstructural analysis (Fig. 6a) showed the overall presence of a single α-Ti phase in the Cp-Ti sample with peaks at 35.3°, 38.6°, 40.5° and minor ones at 2Ɵ angles > 50°. These peaks are still present in the Ti-Cu alloys, however, the intensity diminishes with increased copper content. Notably, peaks at 39.5° and 43.5° become more prominent, suggesting the presence of Ti_2_Cu and unalloyed pure copper, in line with the heterogeneity observed in Figs. 4 and 5. When comparing the XRD scans, it is possible to observe a broadening of the peaks corresponding to α-Ti, Ti_2_Cu and α-Ti at 38.6°, 39.5° and 40.5° as the copper content increases, evident for designed Ti-15Cu and Ti-20Cu. This could be caused by the rapid localised heating and cooling cycles common to PBF-LB technologies, enabling the formation of more refined microstructures and potentially suppressing grain coarsening [48–50].

Mechanical properties were evaluated using microhardness and non-destructive ultrasound probes with copper content substantially influencing performance. Vickers microhardness (Fig. 6b) of Cp-Ti, 199.24 ± 5.24 HV, significantly (*p* < 0.05) increased through the addition of copper, 304.08 ± 15.63 HV, 374.84 ± 24.23 HV and 409.52 ± 13.78 HV, for designed 5, 10 and 15 wt% Cu, respectively, with further alloying leading to a slightly harder material, 428.53 ± 26.75 HV (designed 20 wt%). This effect seems to be caused by lighter Cu-rich precipitates with significantly higher hardness (*p* < 0.05) than those of darker Ti-rich areas. In contrast, longitudinal and shear moduli (Fig. 6c) revealed a significant (*p* < 0.05) decrease from pure titanium, 166.59 ± 4.95 GPa and 45.94 ± 1.51 GPa, respectively. In the case of the longitudinal moduli this difference is relevant for copper content over 5 wt%, stabilising at ~ 149 GPa for designed 15 wt% copper, while copper alloying significantly (*p* < 0.05) affected the shear modulus for all concentrations reaching a minimum for the highest copper content, designed 20 wt% at 36.25 ± 1.15 GPa. It should be noted that the moduli obtained represent the effective properties of the as-built material, inherently incorporating the influence of any defects. However, their potential impact should be limited with prior studies on additively manufactured aluminium alloys [51, 52] showing that 1% volumetric porosity leads to a reduction of about ~ 1.7% in the longitudinal moduli, an order of magnitude below the compositionally driven variations measured here.

**Fig. 7:**
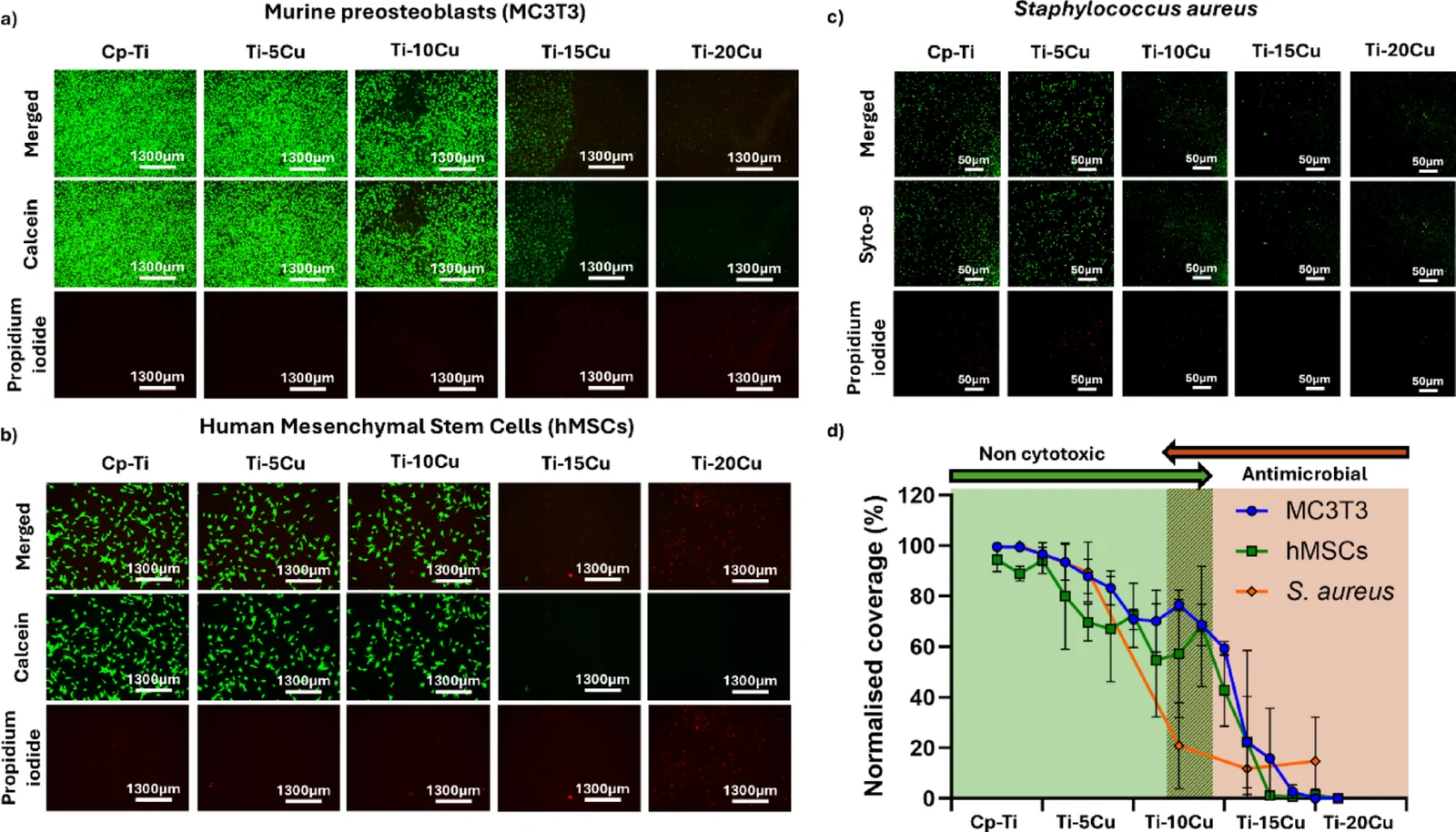
Live/Dead imaging of **(a)** murine MC3T3 preosteoblasts, **(b)** human Mesenchymal Stem Cells (hMSCs) and **(c)** Gram-positive *S. aureus* to create **(d)** design space map for the selection of highly antimicrobial and low cytotoxicity regions to expedite alloy refinement. Captions indicate designed Ti-XCu alloys for X = 0–20 wt%

To enable efficient identification of biologically favourable alloys, Live/Dead images of murine pre-osteoblasts, MC3T3, human Mesenchymal Stem Cells, hMSCs and a gram-positive bacterium, *S. aureus*, were taken across the multi-material sample (Fig. 7a–c). Eukaryotic cells revealed continuous coverage reduction as the copper content increased, achieving a cytotoxic effect (viability < 70% of the control) [53] at the edge of designed Ti-15Cu. For MC3T3 cells, the decrease in viability is lower than that of hMSCs, with inferior coverage observed across the designed 10 wt% copper sample. However, no statistical difference (*p* < 0.05) could be seen with Cp-Ti, suggesting designed Ti-10Cu as the safety breakpoint of the Ti-Cu system. On the other hand, only copper concentrations higher than 5 wt% showcased antimicrobial effectiveness with a limited ~ 10.4% reduction of bacterial coverage displayed by Ti-5Cu when compared to Cp-Ti. By using these results, it was possible to rapidly develop a map (Fig. 7d) to compare both antimicrobial effectiveness and cytocompatibility, revealing Ti-10Cu as the most promising region for future optimisation.

## Discussion

Historically, epitaxial growth and highly directional mechanical properties have been some of the main barriers for metal AM implementation [54]. In this regard, copper is a eutectoid forming element in titanium binary alloys capable of controlling the supercooling region to promote equiaxial growth [33]. Careful control of copper content and thermal history can be used to stimulate Ti_2_Cu nanocrystal nucleation, β grain refinement and prevent martensite coarsening in PBF-LB [34], resulting in enhanced mechanical [55], wear [38] and corrosion [40] resistance. Coupled with its inherent antimicrobial effectiveness and biological activity [4], these benefits make copper a strategic element for PBF-LB across sectors, nevertheless, microstructural optimisation is still an onerous experimental process. This study showcases the potential of an alloy dosing kit to produce a model Ti-Cu multi-material sample using a production PBF-LB machine. Results indicated successful manufacture of a multi-material part dominated by α-Ti with a potential increase of Ti_2_Cu as copper content rose, common in Ti-Cu alloys below 40 wt% copper [42]. Compositional variations revealed a profound influence on mechanical behaviour with higher copper content significantly (*p* < 0.05) enhancing hardness (~ 200, ~ 375 and 429 HV for designed 0, 10 and 20 wt% Cu, Fig. 6). This is in line with the microhardness trends reported by Yi et al. [35] and Ju et al. [38] in the Ti-XCu (217 to 344 HV for 0 to 10 wt% Cu) and Ti-6Al-4V-XCu (315 to 597 HV for 0 to 10 wt% Cu) systems manufactured through arc-melting and PBF-LB, respectively. Previous studies of casted [36] and arc-melted [35] Ti-Cu alloys, however do not indicate a direct correlation between copper content and Young’s/shear modulus values, ~ 105–120 GPa and ~ 41–47 GPa, contrasting with the reduction observed in this study (Fig. 6c). Such divergence may be due to production of the multi-material samples via in-situ alloying, known to be associated with less refined microstructures [19]. Still, it must be recognised that copper content may not be the only driving force behind the trends observed as the increasing instability and defects could have influenced the physicochemical and biological measurements.

Processing of copper via PBF-LB creates challenges given its high laser reflectivity, thermal conductivity, high density, viscosity and lower solidification, which can result in inhomogeneous powder melting and cracking [22, 39] or selective evaporation when mixed with higher boiling point elements, such as titanium [56–58]. Thereby explaining the surface defects, microstructure and discrepancies in designed and measured Ti-Cu composition of the sample (Fig. 4). Green lasers can facilitate copper processing [59], however, recent work in PBF-LB Ti-XCu [33, 39, 40, 55] and Ti-6Al-4V-5Cu [38, 60–62] has indicated that processing control can refine these alloys. In the present work, a volumetric energy density (VED) of 61.73 J/mm^3^ was applied, suggested by Mosallanejad et al. [39] as the minimum intensity to process Ti-5Cu. Nevertheless, the inhomogeneity and cracking observed as copper content increases indicates further parametric optimisation is needed, driven by the large VED required to process pure copper, 667 J/mm^3^, against conventional Ti64, 56 J/mm^3^ [60] and balanced with further addition of copper or alternative rescanning strategies to limit evaporation [57]. This emphasizes the role of the presented approach as a design space downsizing tool with the final material properties of the most promising candidate, i.e. designed Ti-10Cu, still subjected to further parametric and process optimisation. For example, in situ mixing of Titanium and Copper beyond 10 wt% can led to the formation of finer particulates and clustering [43], potentially affecting flowability and consolidation. Thus, processing of these multi-materials to obtain representative properties should consider PBF-LB parameter fine tuning across compositions and variations induced by in situ element mixing, which would be a natural evolution of the presented proof-of-concept where variations in composition and VED could be incorporated in a single geometry.

Beyond mechanical behaviour, the proposed approach can be leveraged to assess other functional properties such as biological outcomes. Specifically, the use of high-throughput in-vitro assays resulted in designed Ti-10Cu being quickly identified as a compromise between cytocompatibility and antibacterial activity (Fig. 7). This is consistent with available research on conventionally manufactured Ti-Cu where recovery and counting assays have repeatedly highlighted 5 wt% copper as their antimicrobial breakpoint [37, 41]. Moreover, MC3T3 pre-osteoblasts and hMSCs were directly seeded onto the multi-material specimens with ≥ 15% copper identified as the cytotoxicity breakpoint, nevertheless, other authors have reported non-cytotoxicity up to Ti-25Cu [37, 41, 63]. This discrepancy can be explained by a preference of assaying Ti-Cu extracts [61, 63] compared with the direct contact methods used herein. In contrast, Rabbitt et al. [42] recently demonstrated detrimental effects of arc-melted Ti-11.5Cu on directly seeded MC3T3. Overall, the Ti-Cu biological trends observed are comparable to other manufacturing methods, suggesting the alloy discovery potential of this approach for bioinspired alloy candidates. While a step in the right direction, it should be mentioned that further work with more functional and long-term biological assays would need to be developed to validate this proof-of-concept approach. For instance, it is fundamental that orthopaedic biomaterials do not detrimentally affect osteoblast and stem cell proliferation, collagen production and differentiation while prokaryotic analysis should be extended to include biofilm formation assays. All these processes take place over longer periods of time than the 24 h in vitro model used in this study. For instance, initial extracellular matrix deposition and ALP production commonly start after 3–7 days of contact [64, 65], while biofilm formation requires at least 3 days of culture [66, 67]. Similarly, cytotoxicity is an essential validation tool for biomaterial discovery, thus, the coverage measurements presented in this manuscript should be substantiated with further quantitative metabolic assays. Moreover, to enable the use of this approach as a biologically relevant screening tool future test should include other critical properties (e.g. immunomodulation and mineralisation) [4].

More generally, other physical performance characteristics could be analysed for specific industrial sectors alongside adaptations to enable continuous elemental additions. For example, copper is known to significantly enhance the ultimate tensile and yield strength of Ti-Cu alloys. Liu et al. reported binary Ti-Cu with 2 and 8% copper via PBF-LB with tensile strength of 810 MPa and 1250 MPa, however elongation was substantially reduced from 17% to 2%, respectively [40]. Similarly, corrosion resistance could be compromised due to galvanic effects if copper content rises over 5% [40, 61]. Nevertheless, increased Cu content over this threshold may be desirable for high wear applications with Ti-6Al-4V-XCu alloys displaying finer grains, solid solution and Ti_2_Cu precipitation strengthening [38]. Thus, it becomes apparent that a more comprehensive battery of assays is needed based on the intended application, which would benefit from the ease of forming different geometry test pieces via PBF-LB. For example, inclusion of particular features to facilitate specific assays such as macro-/micropillars or removable taps for mechanical, wear and corrosion assays [68, 69]. On the other hand, creep analysis may be realised through changes in both sample and testing apparatus. A clear example is Nimer et al. [70] who describe a programmable robotic arm with an attachable thermal chamber to mechanically test an array of AMed pillars. Still, this method employs a full AM plate and large distances between pillars for clearance, impractical in biologically driven assays where miniaturisation and microscopy are preferred. In these cases, other alternatives could be explored to facilitate biological outcome optimisation or even enhancing our understanding on the fundamental mechanism driving cell behaviour. For instance, antimicrobial effectiveness is dependent on both contact killing and ion release with composition and microstructure impacting efficacy [42, 71]. The current approach could be modified to enable the adhesion of reservoirs onto each composition for the analysis of eluted ions over time or their effect in bacterial growth, similarly to the method described by Liu et al. [72]. Consequently, further development of the proposed pipeline and geometry should be performed to enable exploration of PBF-LB processing parameters and assessment of application-based properties.

On the other hand, it must be recognised that alternative approaches for the production of multi-materials using AM technologies exist in the market. The most extensively used would be Direct Energy Deposition (DED) where in-situ mixing of feedstock materials can be realised during material deposition [73]. In these technologies, the powder delivery nozzle can be connected to independent feedstock feeders enabling the consolidation of multiple elements in discrete or even continuous gradients, with available literature displaying its benefits for the selective alloying of titanium, aluminium, nickel, cobalt or iron-based alloys [73, 74]. For example, the system described by Jacquier et al. [75] incorporated four different feeders to enable the deposition of tungsten into copper, aided by chromium and nickel interphases. Such an approach could ease production, while facilitating multi-material development for alloy discovery applications. Nevertheless, the higher resolution (50 vs. 500 μm) and market size, ~ 50.7% in 2024, of PBF-LB technologies [13–15], make them especially attractive for the leveraging of existing infrastructure, while the increased cooling rates of PBF-LB (i.e. up to ~ 10^3^ times that of DED) [76] could be of interest for microstructural refinement such as in the Ti-Cu system.

Finally, this manufacturing approach has been centred on its potential use in alloy discovery, however, it could offer insights into large scale AM processes. For instance, this approach requires halting of the PBF-LB process for material loading, fundamentally affecting the thermal profile. This aspect could be used to mimic unintentional build interruptions (e.g. feedstock topping, power outage, etc.) [77, 78] or planned operations in hybrid AM manufacturing common in aerospace. The described dosing kit may then be used as a quality assurance tool to study the role of restarting the PBF-LB process in defect formation as a result of microstructural and residual stresses, oxidation or part repositioning [79, 80].

## Conclusions

Herein, we propose a novel approach for the manufacture of multi-material samples as a tool for screening novel alloy spaces, enabling down selection of promising compositions for further optimisation and testing. A bespoke alloy dosing kit was developed that combines in-situ alloying with a reduced build volume kit for a production PBF-LB machine. A Ti-Cu case study was used to demonstrate coupling of this approach with high-throughput analysis methods to explore both mechanical and biological performance of the produced multi-material specimens. This approach effectively identified the 10Cu (wt%) compositional area as a region of interest for further onward microstructural and parametric optimisation. Geometry of the multi-materials specimens could also be refined to explore opportunities for future alignment with other characterisation methodology. In summary, we offer a methodology for modified usage of a production PBF-LB machine facilitating multi-material sample fabrication to screen material spaces.

## Data Availability

Data will be made available on request.

## References

[1] Smil V (2013) Making the modern world: materials and dematerialization. Wiley, Hoboken

[2] Zhan L, Kong C, Zhao C, Cui X, Zhang L, Song Y, Lu Y, Xia L, Ma K, Yang H, Shu S, Dong B, Qiu F, Jiang Q (2025) Recent advances on magnesium alloys for automotive cabin components: materials, applications, and challenges. J Mater Res Technol 36:9924–9961

[3] King DJM, Cheung STY, Humphry-Baker SA, Parkin C, Couet A, Cortie MB, Knowles AJ (2019) High temperature, low neutron cross-section high-entropy alloys in the Nb-Ti-V-Zr system. Acta Mater 166:435–446

[4] Rabbitt D, Villapún VM, Carter LN, Man K, Lowther M, O’Kelly P, Knowles AJ, Mottura A, Tang YT, Luerti L, Reed RC, Cox SC (2025) Rethinking biomedical titanium alloy design: a review of challenges from biological and manufacturing perspective. Adv Healthc Mater 14(4):2403129 10.1002/adhm.202403129 PMC11804846 39711273

[5] Pickering EJ, Carruthers AW, Barron PJ, Middleburgh SC, Armstrong DE, Gandy AS (2021) High-entropy alloys for advanced nuclear applications. Entropy 23(1):98 10.3390/e23010098 PMC7827623 33440904

[6] Zheng K, He Z, Che L, Cheng H, Ge M, Si T, Xu X (2024) Deep alloys: metal materials empowered by deep learning. Mater Sci Semicond Process 179:108514

[7] Oh ST, Woo KD, Kim JH, Kwak SM (2017) The effect of Al and V on microstructure and transformation of β phase during solution treatments of cast Ti-6Al-4V alloy. Korean J Met Mater 55(3):150–155

[8] Cherenda NN, Leivi A, Petukh AB, Uglov VV, Grigoriev SN, Vereschaka AA, Astashynski VM, Kuzmitski AM (2024) Modification of Ti-6Al-4V titanium alloy surface relief by compression plasma flows impact. High Temp Mater Process 28(2):7–24

[9] Yu H, Xu W, Van Der Zwaag S (2018) The compositional dependence of the microstructure and properties of CMSX-4 superalloys. Metall Mater Trans A 49(1):406–416

[10] Guo W, Zhao H, Ru Y, Pei Y, Wang J, Liu Q, Li X, wang H, Zhang S, Gong S, Li S (2022) Topologically closed packed phase and its interaction with dislocation movement in Ni–based superalloy during high–temperature creep. Crystals 12(10):1446

[11] Oliveira NT, Aleixo G, Caram R, Guastaldi AC (2007) Development of Ti–Mo alloys for biomedical applications: Microstructure and electrochemical characterization. Mater Sci Eng A 452:727–731

[12] Kuroda PAB, Buzalaf MAR, Grandini CR (2016) Effect of molybdenum on structure, microstructure and mechanical properties of biomedical Ti-20Zr-Mo alloys. Mater Sci Eng C 67:511–515 10.1016/j.msec.2016.05.053 27287149

[13] Forecast MD (2025) Europe additive manufacturing with metal powder market segmented by product, application and region—Market size, growth trends and Y-o-Y forecasts 2025–2033

[14] Babuska TF, Krick BA, Susan DF, Kustas AB (2021) Comparison of powder bed fusion and directed energy deposition for tailoring mechanical properties of traditionally brittle alloys. Manuf Lett 28:30–34

[15] Imran MMA, Che Idris A, De Silva LC, Kim YB, Abas PE (2024) Advancements in 3D printing: directed energy deposition techniques, defect analysis, and quality monitoring. Technologies 12(6):86

[16] Sing SL, Huang S, Goh GD, Goh GL, Tey CF, Tan JHK, Yeong WY (2021) Emerging metallic systems for additive manufacturing: In-situ alloying and multi-metal processing in laser powder bed fusion. Prog Mater Sci 119:100795

[17] Shu T, Yuan J, Liu F, Shen W, Cheng GJ (2025) In-situ alloying and microstructural control in high entropy alloys with nano-carbide-framework via powder bed fusion. Addit Manuf 104:104766

[18] Portoles L, Blasco JR, Martín JM, Burgos N, Borghetto M, Zoz A, Ludwig N, Maccio T, Marcos MD, Garcia-Manrique JA, Solano L (2024) In situ synthesis of titanium alloy powders reinforced with nanoparticles for powder bed fusion: a step towards safer and more sustainable manufacturing. Addit Manuf 82:104032

[19] Xu D, Yang W, Cao P (2025) In situ multi-metal alloying in laser-based additive manufacturing: a concise review. Compos B Eng 299:112443

[20] Mosallanejad MH, Niroumand B, Aversa A, Saboori A (2021) In-situ alloying in laser-based additive manufacturing processes: a critical review. J Alloys Compd 872:159567

[21] Yao L, Ramesh A, Xiao Z, Chen Y, Zhuang Q (2023) Multimetal research in powder bed fusion: a review. Materials 16(12):4287 10.3390/ma16124287 PMC10305177 37374471

[22] Wang D, Liu L, Deng G, Deng C, Bai Y, Yang Y, Wang Y, Lin X, Han C (2022) Recent progress on additive manufacturing of multi-material structures with laser powder bed fusion. Virtual Phys Prototyp 17(2):329–365

[23] Schneck M, Horn M, Schmitt M, Seidel C, Schlick G, Reinhart G (2021) Review on additive hybrid-and multi-material-manufacturing of metals by powder bed fusion: state of technology and development potential. Prog Addit Manuf 6(4):881–894

[24] Mehrpouya M, Tuma D, Vaneker T, Afrasiabi M, Bambach M, Gibson I (2022) Multimaterial powder bed fusion techniques. Rapid Prototyp J 28(11):1–19

[25] Demir AG, Previtali B (2017) Multi-material selective laser melting of Fe/Al-12Si components. Manuf Lett 11:8–11

[26] Wei C, Li L, Zhang X, Chueh YH (2018) 3D printing of multiple metallic materials via modified selective laser melting. CIRP Ann Manuf Technol 67(1):245–248

[27] Wits WW, Amsterdam E (2021) Graded structures by multi-material mixing in laser powder bed fusion. CIRP Ann Manuf Technol 70(1):159–162

[28] Demir AG, Kim J, Caltanissetta F, Hart AJ, Tasan CC, Previtali B, Colosimo BM (2022) Enabling multi-material gradient structure in laser powder bed fusion. J Mater Process Technol 301:117439

[29] Wang D, Deng GW, Yang YQ, Chen J, Wu WH, Wang HL, Tan CL (2021) Interface microstructure and mechanical properties of selective laser melted multilayer functionally graded materials. J Cent South Univ 28(4):1155–1169

[30] Maskery I, Aboulkhair NT, Aremu AO, Tuck CJ, Ashcroft IA, Wildman RD, Hague RJM (2016) A mechanical property evaluation of graded density Al-Si10-Mg lattice structures manufactured by selective laser melting. Mater Sci Eng A 670:264–274

[31] Liu Z, Zhou Q, Liang X, Wang X, Li G, Vanmeensel K, Xie J (2024) Alloy design for laser powder bed fusion additive manufacturing: a critical review. Int J Extreme Manuf 6(2):022002

[32] Luo L, Zhong Q, Chen ZQ, Wu XH, Li SM, Xue ZZ, Lu YJ, Luo K, Zhao W (2025) 3D-printed copper-containing tailored titanium alloys with corrosion resistance, biocompatibility, and anti-inflammatory properties for enhanced guided bone regeneration. Front Bioeng Biotechnol 13:1647678 10.3389/fbioe.2025.1647678 PMC12408666 40918437

[33] Zhang D, Qiu D, Gibson MA, Zheng Y, Fraser HL, StJohn DH, Easton MA (2019) Additive manufacturing of ultrafine-grained high-strength titanium alloys. Nature 576(7785):91–95 10.1038/s41586-019-1783-1 31802014

[34] Liu H, Wang H, Ren L, Qiu D, Yang K (2023) Antibacterial copper-bearing titanium alloy prepared by laser powder bed fusion for superior mechanical performance. J Mater Sci Technol 132:100–109

[35] Yi C, Ke Z, Zhang L, Tan J, Jiang Y, He Z (2020) Antibacterial Ti-Cu alloy with enhanced mechanical properties as implant applications. Mater Res Express 7(10):105404

[36] Kikuchi M, Takada Y, Kiyosue S, Yoda M, Woldu M, Cai Z, Okuno O, Okabe T (2003) Mechanical properties and microstructures of cast Ti–Cu alloys. Dent Mater 19(3):174–181 10.1016/s0109-5641(02)00027-1 12628428

[37] Celles CAS, dos Reis AC (2025) Effect of copper incorporated into titanium (cp-Ti) and its alloys (Ti-6Al-4V) and its promising antibacterial effect: a systematic review. Dent Rev 5:100156

[38] Ju J, Zan R, Shen Z, Wang C, Peng P, Wang J, Sun B, Xiao B, Li Q, Liu S, Yang T (2023) Remarkable bioactivity, bio-tribological, antibacterial, and anti-corrosion properties in a Ti-6Al-4V-xCu alloy by laser powder bed fusion for superior biomedical implant applications. Chem Eng J 471:144656

[39] Mosallanejad MH, Niroumand B, Aversa A, Manfredi D, Saboori A (2021) Laser powder bed fusion in-situ alloying of Ti-5% Cu alloy: process-structure relationships. J Alloys Compd 857:157558

[40] Liu H, Zhang X, Shi L, Hong Y, Yao H, Ren L, Yang K (2024) Effect of Cu content on the properties of laser powder bed fused biomedical titanium alloy. J Mater Res Technol 30:8992–8998

[41] Alshammari Y, Elkork N, Moussa L, Esmaeil F, Saeed M, Alsarraf M, Alfarhan A, Alrashidi MA, Bolzoni L (2025) Systematic review of metal-based alloys with autogenous antibacterial capability. Crit Rev Solid State Mater Sci 50:1–48

[42] Rabbitt D, O’Kelly P, Villapún VM, Carter LN, Knowles AJ, Cox SC (2025) Antimicrobial titanium–copper alloys: the role of microstructure in arc-melted compositions. Adv Eng Mater 27(16):2500347

[43] Rabbitt D (2025) Development of antimicrobial metal alloys chemical engineering. University of Birmingham

[44] Carter LN, Villapún VM, Andrews J, Grandjean TR, Dardis J, Cox SC (2024) Modelling process monitoring data in laser powder bed fusion: A pragmatic route to additive manufacturing quality assurance. Addit Manuf Lett 11:100252

[45] Huang M, Sha G, Huthwaite P, Rokhlin SI, Lowe MJS (2021) Longitudinal wave attenuation in polycrystals with elongated grains: 3D numerical and analytical modelin. J Acoust Soc Am 149(4):2377–2394 10.1121/10.0003955 33940885

[46] Huang M, Huthwaite P, Rokhlin SI, Lowe MJ (2022) Finite-element and semi-analytical study of elastic wave propagation in strongly scattering polycrystals. Proc R Soc A 478:20210850 10.1098/rspa.2021.0850 PMC8848240 35221773

[47] Kalienko MS, Zhelnina AV, Volkov AV, Leder MO, Tolstykh NA, Bocharov AI (2020) A New approach to estimation of the thermal conductivity of titanium alloys. Crystallogr Rep 65(6):844–848

[48] Thampy V, Fong AY, Calta NP, Wang J, Martin AA, Depond PJ, Kiss AM, Guss G, Xing Q, Ott RT, van Buuren A, Toney MF, Weker JN, Kramer MJ, Matthews MJ, Tassone CJ, Stone KH (2020) Subsurface cooling rates and microstructural response during laser based metal additive manufacturing. Sci Rep 10(1):1981 10.1038/s41598-020-58598-z PMC7005153 32029753

[49] Kosiba K, Gustmann T, Kim JT, Seok J, Jung J, Beyer L, Scudino S, Giebeler L, Han J, Hufenbach JK (2023) Experimental cooling rates during high-power laser powder bed fusion at varying processing conditions. J Alloys Comp 967:171773

[50] Hooper PA (2018) Melt pool temperature and cooling rates in laser powder bed fusion. Addit Manuf 22:548–559

[51] Ni K, Li Q, Huang M, Liu Y (2026) Ultrasonic characterisation of process-induced pores in selective laser melted TiB2/Al composites. NDT E Int 160:103629

[52] Liu Y, Li Q, Ni K, Huang M (2026) Three-dimensional simulation and experimental investigation of ultrasound propagation through pores in additively manufactured metals. Ultrasonics 164:108000 10.1016/j.ultras.2026.108000 41713378

[53] Thangaraju P, Varthya SB (2022) ISO 10993: biological evaluation of medical devices. In: Medical device guidelines and regulations handbook. Springer, Cham

[54] Ni C, Zhu J, Zhang B, An K, Wang Y, Liu D, Lu W, Zhu L, Liu C (2025) Recent advance in laser powder bed fusion of Ti–6Al–4V alloys: microstructure, mechanical properties and machinability. Virtual Phys Prototyp 20(1):e2446952

[55] Wang SX, Li SF, Misra RDK, Kondoh K, Kariya S, Yang YF (2025) Achieving strong and ductile as-printed Ti-Cu alloys via tailoring eutectoid morphology. Mater Res Lett 13(6):577–586

[56] Huber F, Bartels D, Schmidt M (2021) In-situ alloy formation of a WMoTaNbV refractory metal high entropy alloy by laser powder bed fusion (PBF-LB/M). Materials 14(11):3095 10.3390/ma14113095 PMC8201384 34200096

[57] Strauch AL, Altmann ML, Toenjes A (2026) In situ alloying from powder blends enables processing of high copper content Ti-6Al-4V by laser powder bed fusion. Discov Mater 6(1):184

[58] Steinmeier P, Hoyer KP, Lopes Dias NF, Zielke R, Tillmann W, Schaper M (2025) In situ alloying of Ti-6Al-7Nb with copper using laser powder bed fusion. Crystals 15(12):1053

[59] Ning Z, Hu H, Zhao T, Wang S, Song M (2024) Enhanced electrical and mechanical properties of additively manufactured pure copper with green laser. J Mater Process Technol 334:118615

[60] Goettgens VS, Kaserer L, Braun J, Letofsky-Papst I, Mitsche S, Leichtfried G (2023) Microstructure of a modulated Ti-6Al-4V–Cu alloy fabricated via in situ alloying in laser powder bed fusion. Materialia 28:101731

[61] Guo S, Lu Y, Wu S, Liu L, He M, Zhao C, Gan Y, Lin J, Luo J, Xu X, Lin J (2017) Preliminary study on the corrosion resistance, antibacterial activity and cytotoxicity of selective-laser-melted Ti6Al4V-xCu alloys. Mater Sci Eng C 72:631–640 10.1016/j.msec.2016.11.126 28024632

[62] Chen Y, Yang W, Zhu S, Shi Y (2023) Microstructural, mechanical and in vitro biological properties of Ti6Al4V-5Cu alloy fabricated by selective laser melting. Mater Charact 200:112858

[63] Zhang E, Zheng L, Liu J, Bai B, Liu C (2015) Influence of Cu content on the cell biocompatibility of Ti–Cu sintered alloys. Mater Sci Eng C 46:148–157 10.1016/j.msec.2014.10.021 25491971

[64] Quarles LD, Yohay DA, Lever LW, Caton R, Wenstrup RJ (1992) Distinct proliferative and differentiated stages of murine MC3T3-E1 cells in culture: an in vitro model of osteoblast development. J Bone Min Res 7(6):683–692 10.1002/jbmr.5650070613 1414487

[65] Wang L, Ruan M, Bu Q, Zhao C (2025) Signaling pathways driving MSC osteogenesis: mechanisms, regulation, and translational applications. Int J Mol Sci 26(3):1311 10.3390/ijms26031311 PMC11818554 39941080

[66] O’Toole G, Kaplan HB, Kolter R (2000) Biofilm formation as microbial development. Annu Rev Microbiol 54(1):49–79 10.1146/annurev.micro.54.1.49 11018124

[67] Sauer K, Stoodley P, Goeres DM, Hall-Stoodley L, Burmølle M, Stewart PS, Bjarnsholt T (2022) The biofilm life cycle: expanding the conceptual model of biofilm formation. Nat Rev Microbiol 20(10):608–620 10.1038/s41579-022-00767-0 PMC9841534 35922483

[68] Zhang X, Guan K, Zhong J, Chen G, Wang W, Wang Q (2021) A macro-pillar compression technique for determining true stress-strain curves of steels. Measurement 168:108407

[69] Taufiqurrakhman M, Khan T, Bryant MG (2024) Investigating the roles of protein on the cobalt alloy surface degradation for biomedical implant through tribocorrosion mechanisms. Tribol Lett 72(2):54

[70] Nimer SM, Gienger IV EB, Storck SM, Lennon AM (2024) Autonomous mechanical performance characterization of three-dimensional articles on a build plate

[71] Wang Y, Wen T, Mao F, Yang S, Zhang Q, Fu X, Zhai C, Zhang H (2025) Engineering copper and copper-based materials for a post-antibiotic era. Front Bioeng Biotechnol 13:1645362 10.3389/fbioe.2025.1644362 PMC12364917 40843444

[72] Liu Y, Padmanabhan J, Cheung B, Liu J, Chen Z, Scanley BE, Wesolowski D, Pressley M, Broadbridge CC, Altman S, Schwarz UD, Kyriades TR, Schroers J (2016) Combinatorial development of antibacterial Zr-Cu-Al-Ag thin film metallic glasses. Sci Rep 6(1):26950 10.1038/srep26950 PMC4882501 27230692

[73] Feenstra DR, Banerjee R, Fraser HL, Huang A, Molotnikov A, Birbilis N (2021) Critical review of the state of the art in multi-material fabrication via directed energy deposition. Curr Opin Solid State Mater Sci 25(4):100924

[74] Ahn DG (2021) Directed energy deposition (DED) process: state of the art. Int J Precis Eng Manuf - Green Technol 8(2):703–742

[75] Jacquier V, Zollinger J, Zeller P, Schuster F, Maskrot H, Pacquentin W (2024) Directed energy deposition with a graded multi-material compatibility interface enables deposition of W on Cu. Mater Des 244:113114

[76] Chechik L, Todd I (2023) Inconel 718 two ways: powder bed fusion vs. directed energy deposition. Addit Manuf Lett 6:100145

[77] Stokes RM, Yadollahi A, Priddy MW, Bian L, Hammond VH, Doude HR (2023) Effects of build interruption and restart procedure on microstructure and mechanical properties of laser powder bed fusion Al-Si-10Mg. J Mater Eng Perform 32(4):1576–1588

[78] Terrazas-Najera CA, Mayoral FL, Garcia OF, Hossain MS, Espalin D, Fernandez A, Murr LE, Wicker RB (2021) Effects of process interruptions on microstructure and mechanical properties of three face centered cubic alloys processed by laser powder bed fusion. J Manuf Process 66:397–406

[79] Penchev P, Bhaduri D, Carter L, Mehmeti A, Essa K, Dimov S, Adkins NJ, Maillol N, Bajolet J, Maurath J, Jurdeczka U (2019) System-level integration tools for laser-based powder bed fusion enabled process chains. J Manuf Syst 50:87–102

[80] Mahtabi M, Yadollahi A, Stokes R, Doude H, Priddy M (2023) Effect of build interruption during laser powder bed fusion process on structural integrity of Ti-6Al-4V. Eng. Fail Anal 153:107626

